# Genetic Polymorphisms of lncRNA *LINC00673* as Predictors of Hepatocellular Carcinoma Progression in an Elderly Population

**DOI:** 10.3390/ijms232112737

**Published:** 2022-10-22

**Authors:** Lan-Ting Yuan, Yi-Chieh Yang, Hsiang-Lin Lee, Pei-Chun Shih, Li-Hsin Chen, Chih-Hsin Tang, Lun-Ching Chang, Hsiang-Ling Wang, Shun-Fa Yang, Ming-Hsien Chien

**Affiliations:** 1 Department of Internal Medicine, Division of Hepatology and Gastroenterology, Yuan’s General Hospital, Kaohsiung 802, Taiwan; 2 Graduate Institute of Clinical Medicine, College of Medicine, Taipei Medical University, Taipei 110, Taiwan; 3 Department of Medical Research, Tungs’ Taichung MetroHarbor Hospital, Taichung 433, Taiwan; 4 School of Medicine, Chung Shan Medical University, Taichung 402, Taiwan; 5 Department of Surgery, Chung Shan Medical University Hospital, Taichung 402, Taiwan; 6 Department of Laboratory Medicine, National Taiwan University Hospital, Taipei 100, Taiwan; 7 School of Medicine, China Medical University, Taichung 404, Taiwan; 8 Chinese Medicine Research Center, China Medical University, Taichung 404, Taiwan; 9 Department of Medical Laboratory Science and Biotechnology, College of Medical and Health Science, Asia University, Taichung 413, Taiwan; 10 Department of Mathematical Sciences, Florida Atlantic University, Boca Raton, FL 33431, USA; 11 Department of Beauty Science, National Taichung University of Science and Technology, Taichung 404, Taiwan; 12 Institute of Medicine, Chung Shan Medical University, Taichung 402, Taiwan; 13 Department of Medical Research, Chung Shan Medical University Hospital, Taichung 402, Taiwan; 14 TMU Research Center of Cancer Translational Medicine, Taipei Medical University, Taipei 100, Taiwan; 15 Pulmonary Research Center, Wan Fang Hospital, Taipei Medical University, Taipei 100, Taiwan; 16 Traditional Herbal Medicine Research Center, Taipei Medical University Hospital, Taipei 100, Taiwan

**Keywords:** long noncoding RNA, *long intergenic noncoding RNA 00673*, single-nucleotide polymorphism, hepatocellular carcinoma, susceptibility, progression

## Abstract

Long noncoding (lnc)RNAs are reported to be key regulators of tumor progression, including hepatocellular carcinoma (HCC). The lncRNA *long intergenic noncoding RNA 00673 (LINC00673)* was indicated to play an important role in HCC progression, but the impacts of genetic variants (single-nucleotide polymorphisms, SNPs) of *LINC00673* on HCC remain unclear. A TaqMan allelic discrimination assay was performed to analyze the genotypes of three tagging SNPs, viz., rs9914618 G > A, rs6501551 A > G, and rs11655237 C > T, of *LINC00673* in 783 HCC patients and 1197 healthy subjects. Associations of functional SNPs of *LINC00673* with HCC susceptibility and clinicopathologic variables were analyzed by logistic regression models. After stratification by confounding factor, we observed that elderly patients (≥60 years) with the *LINC00673* rs9914618 A allele had an increased risk of developing HCC under a codominant model (*p* = 0.025) and dominant model (*p* = 0.047). Moreover, elderly patients carrying the GA + AA genotype of rs9914618 exhibited a higher risk of having lymph node metastasis compared to those who were homozygous for the major allele (*p* = 0.013). Genotype screening of rs9914618 in HCC cell lines showed that cells carrying the AA genotype expressed higher *LINC00673* levels compared to the cells carrying the GG genotype. Further analyses of clinical datasets from the Cancer Genome Atlas (TCGA) showed that *LINC00673* expressions were upregulated in HCC tissues compared to normal tissues, and were correlated with advanced clinical stages and poorer prognoses. In conclusions, our results suggested that the *LINC00673* rs9914618 polymorphism may be a promising HCC biomarker, especially in elderly populations.

## 1. Introduction

Hepatocellular carcinoma (HCC), accounting for over 90% of all primary hepatic malignancies, is ranked as the fourth leading cause of cancer deaths globally [1]. HCC initiation has been majorly correlated with environmental and genetic factors. For example, obesity, aflatoxin intake, alcohol and cigarette consumption, and hepatitis B or C virus (HBV or HCV) infection are usually recognized as environmental factors contributing to HCC development [1]. The HCC incidence varies geographically. In Taiwan, HBV infection is still a major risk factor for HCC patients [2]. As for genetic factors, emerging single-nucleotide polymorphisms (SNPs) in some noncoding genes such as micro (mi)RNA and long noncoding (lnc)RNAs were reported to be associated with HCC risk, development, and prognosis [3,4,5]. SNPs are some of the most common heritable mutations that induce DNA sequence polymorphisms at the gene level and were shown to have potential value for HCC prediction.

LncRNAs are a novel class of noncoding RNAs (>200 nt) which were shown to play significant roles in influencing the tumorigenesis, metastasis, and prognosis of cancers [6]. Recently, genome-wide association studies (GWASs) showed that only a very small portion of SNPs associated with complex diseases such as cancers was located in protein-coding regions. In contrast, the remaining part (>90%) was located in noncoding regions [7,8]. To the present, there are still few studies that focused on lncRNA polymorphisms as predictive biomarkers for HCC risk and progression.

*LINC00673*, also known as *SRA-like noncoding RNA (SLNCR)*, is an lncRNA located on chromosome 17q24.3. Based on the Ensembl genome browser, the locus of *LINC00673* largely overlaps with the locus of *LINC00511*, and 106 transcripts of *LINC00673* were found to be derived from *LINC00511*, including *LINC00673-v1-5* [9]. Recent studies indicated that *LINC00673* acts as an oncogene or tumor suppressor gene in the occurrence and development of several cancer types including gastric [10], breast [11], pancreatic [12], lung [13], and prostate cancers [14]. In HCC, *LINC00673* was reported to compete with and absorb *miR-205* and promote progression of HCC [15]. As to the impact of *LINC00673* SNPs on cancer, current studies mainly focused on rs11655237, a common noncoding transcript variant of *LINC00673*. This SNP was shown to increase the susceptibility of populations to gastric cancer [16], cervical cancer [17], pancreatic cancer [18], and neuroblastomas [19]. Moreover, the rs11655237 polymorphism was reported to be correlated with a risk of hepatoblastomas (HBs), the most common childhood hepatic malignancy, in Chinese children [20]. However, the roles of functional SNPs of *LINC00673* within the context of HCC in adult populations have not yet been investigated.

Herein, rs11655237, together with two SNPs (rs6501551 and rs9914618) located in *LINC00673* with a RegulomeDB score of <3, were chosen as tagSNPs. We investigated their associations with the risk and clinical characteristics of HCC in an adult Taiwanese population sample.

## 2. Results

### 2.1. Study Population Characteristics

Demographic characteristics of recruited subjects are shown in Table 1. This study group comprised 783 pathologically confirmed HCC patients (542 males and 241 females) and 1197 cancer-free controls (837 males and 360 females). No significant differences between HCC patients and healthy controls were observed in terms of the distributions of age <60 and ≥60 years (*p* = 0.383), gender (*p* = 0.739), or smoking status (*p* = 0.284). Consistent with findings from other studies [21], significantly higher frequencies of HCC patients, compared to healthy controls, had a habit of alcohol consumption (14.1% vs. 35%; *p* < 0.001) and were positive for the hepatitis B surface antigen (HBsAg) (12.2% vs. 34%; *p* < 0.001). In HCC patients, higher proportions were diagnosed as being at early clinical (72.8%) and T stages (73.6%), with liver cirrhosis (59%) or without lymph node (97.2%), distal metastasis (96.2%), or vascular invasion (64.1%).

### 2.2. Association Studies of LINC00673 Genetic Polymorphisms and HCC Risks

To investigate possible associations of *LINC00673* gene polymorphisms with the risk of developing HCC, genotype frequencies of selected tagSNPs (rs9914618, rs6501551, and rs11655237) were evaluated in all recruited cohort (Table 2). Genotypic frequencies of these tagSNPs in the healthy control group conformed to Hardy–Weinberg equilibrium (rs9914618: *p* = 0.639; rs6501551: *p* = 0.284; rs11655237: *p* = 0.868). After adjusting for potential confounding factors including age, gender, alcohol consumption, and cigarette smoking, we observed no significant correlations of these *LINC00673* variants with the occurrence of HCC between HCC patients and controls (Table 2). The recruited cohort was further divided by age, and we observed that elderly patients (≥60 years) with the GA/AA genotypes of *LINC00673* rs9914618 had an increased risk of HCC under the codominant model (GA vs. GG: adjusted odds ratio (AOR), 1.328; 95% confidence interval (CI), 1.036–1.703; *p* = 0.025) and dominant model (GA + AA vs. GG: AOR, 1.129; 95% CI, 1.002–1.273; *p* = 0.047) (Table 3).

### 2.3. Relationships of LINC00673 Genetic Polymorphisms with Clinicopathological Features in HCC Patients

To further investigate the impacts of *LINC00673* genetic polymorphisms on HCC progression, several clinicopathological features such as primary tumor size, clinical stage, tumor vascular invasion and metastases, hepatitis viral infection, and liver cirrhosis were chosen and are shown in Table 4. We observed that patients carrying at least one minor allele of rs9914618 (GA and AA) were prone to develop lymph node (LN) metastasis, compared to their corresponding wild-type genotype (GG) (OR, 2.346; *p* = 0.073). The HCC population was further divided into younger (<60 years) and elderly (≥60 years) groups and differences between *LINC00673* SNPs and HCC in clinicopathological features were determined for these two groups. The results showed only elderly HCC patients harboring at least one minor allele of rs9914618 (GA and AA) had a significantly (*p* = 0.013) 3.970-fold higher risk (95% CI, 1.227–12.845) of developing LN metastasis compared to those homologous to the major allele (Table 5).

### 2.4. Upregulation of LINC00673 Is Observed in HCC Tissues and Correlated with Tumor Progression and a Poor Prognosis

**Figure 1:**
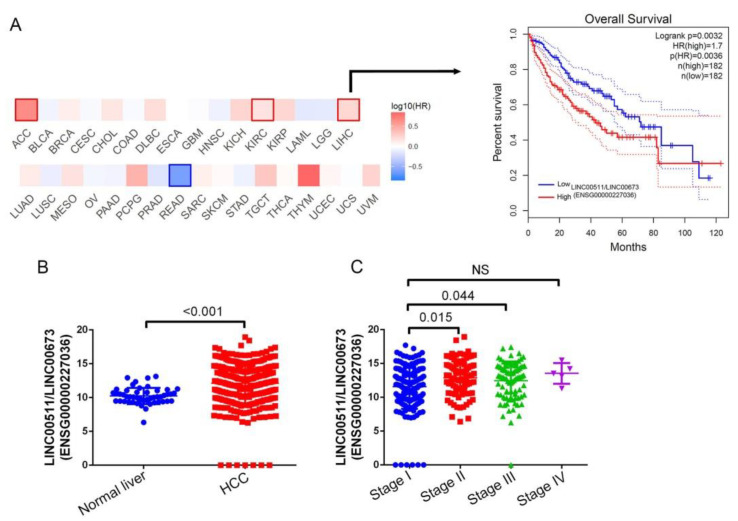
*ENSG00000227036* is highly expressed in hepatocellular carcinoma (HCC) and is associated with a poor prognosis. (**A**) Survival heat map of hazard ratios (HRs) showing the prognostic impacts of *ENSG00000227036* on multiple cancer types (left panel). Kaplan–Meier curves for overall survival of patients with HCC, as categorized according to high or low expression of *ENSG00000227036*. The *p* value indicates a comparison between patients with *ENSG00000227036*^high^ (red color line) and *ENSG00000227036*^low^ (blue color line) (right panel). Data in both the left and right panels are from available online GEPIA2 databases. (**B**) Expression levels of the *ENSG00000227036* gene in normal and HCC tissues were compared according to data from TCGA datasets. (**C**) *ENSG00000227036* gene expression levels in HCC from TCGA were compared according to the clinical stage. Statistical significance of results from B and C were analyzed by a *t* test. *p* < 0.05 was considered significant. NS—not significant.

Considering the potential effects of *LINC00673* polymorphic genotypes on *LINC00673* expression levels [18], correlations of *LINC00673* expression levels with clinical significance and survival rates in HCC patients were further analyzed by examining cases of HCC from the TCGA dataset. According to the GEPIA2 website, we observed the prognostic value of *LINC00673* in 33 different cancer types and found that high expression of *LINC00673* showed poor prognostic impacts on four cancer types, including adrenocortical carcinoma (ACC), kidney renal clear cell carcinoma (KIRC), thymoma (THYM), and HCC (Figure 1A, left panel) and Kaplan–Meier curves for overall survival (OS) of patients with HCC are shown in the right panel of Figure 1A. We also observed significantly higher *LINC00673* transcripts in HCC compared to noncancerous tissues (Figure 1B). Furthermore, patients with advanced clinical stages (II or III) showed significantly higher *LINC00673* expression in tumors compared to patients at an early clinical stage (I) (Figure 1C). The clinical data mentioned above suggest that *LINC00673* genetic variants may affect *LINC00673* expression levels and subsequently modulate the formation and progression of HCC.

### 2.5. The Correlations of LINC00673 Genetic Variants with LINC00673 Expression Levels

**Figure 2:**
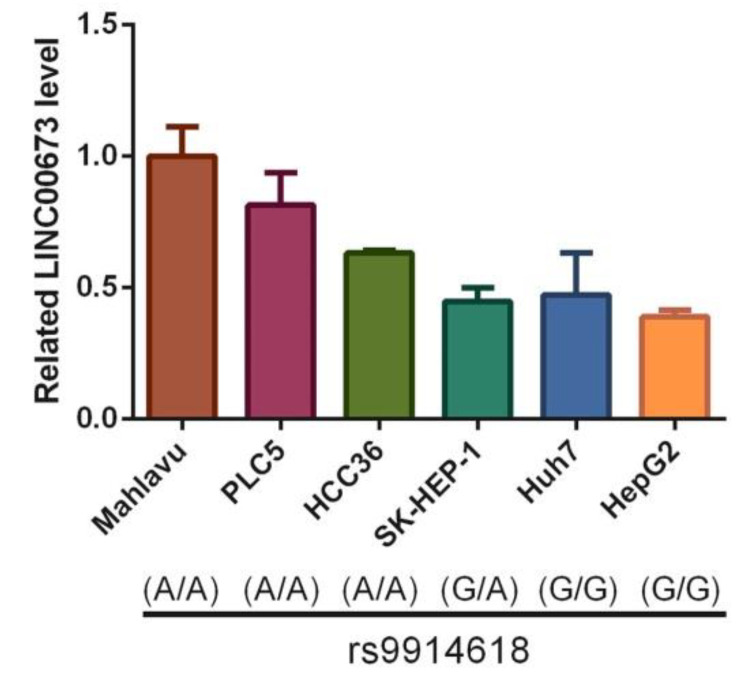
Correlations of *LINC00673* rs9914618 genotypes with *LINC00673* levels in six hepatocellular carcinoma (HCC) cell lines. Lower panel: *LINC00673* rs9914618 genotypes in HCC cells (Mahlavu, PLC5, HCC36, SK-HEP-1, Huh7, and HepG2) were detected by a TaqMan SNP Genotyping Assay. Upper panel: expression levels of *LINC00673* were determined by an RT-qPCR.

We next examined the correlations between *LINC00673* rs9914618 genotypes and *LINC00673* expression levels among six HCC cell lines (Mahlavu, PLC5, HCC36, SK-HEP-1, Huh7, and HepG2). We observed that Mahlavu, PLC5, and HCC36 cells carried the AA genotype of rs9914618 compared to Huh7 and HepG2 cells, which carried the GG genotype (Figure 2, lower panel). From the results of RT-qPCR, we found that Mahlavu, PLC5, and HCC36 cells harboring the AA genotype expressed higher *LINC00673* levels than Huh7 and HepG2 cells harboring the GG genotype (Figure 2, upper panel).

## 3. Discussion

Although HCC is recognized as one of the most prevalent forms of cancer, but the pathophysiology and underlying causes of HCC are less well understood. Therefore, identifying useful biomarkers for surveillance and early diagnosis of HCC is still deficient. Serum alpha fetal protein (AFP) is a common and clinically used tumor biomarker for HCC surveillance; however, recent reports indicated that the specificity and sensitivity of AFP for early diagnosis of HCC are not satisfactory [22]. Thus, there is still a need to search for novel biomarkers for early HCC detection. Accumulating evidence has manifested that several serum lncRNAs are potential biomarkers for predicting the occurrence, progression, and prognosis of HCC [23]. For example, Xu et al. found that serum levels of *LINC00635* and *ENSG00000258332.1* were upregulated in HCC patients and correlated with poor prognoses [24]. Wang et al. indicated that serum levels of *lncRNA uc007biz.1 (LRB1)* were positively correlated with tumor stages and negatively associated with OS in patients with HCC [25]. Moreover, Zheng et al. indicated that upregulation of *urothelial cancer-associated (UCA) 1* in serum of HCC patients was associated with advanced TNM stages [26]. *LINC00673* is a recently discovered lncRNA, and the oncogenic roles of *LINC00673* in HCC were previously reported, including functions such as the promotion of proliferation and metastasis of HCC through negatively regulating *miR-205* [15]. In the present study, we also observed that *LINC00673* was upregulated in HCC and correlated with advanced clinical stages and poor prognoses of HCC patients. Although the oncogenic roles of *LINC00673* in HCC have been studied, knowledge of the clinical relevance of *LINC00673* SNPs in HCC, which might affect the functional changes and expression of *LINC00673*, is still lacking. Herein, we first identified that the *LINC00673* SNPs play critical roles in influencing the occurrence and clinicopathological features of HCC in a Taiwanese population.

**Figure 3:**
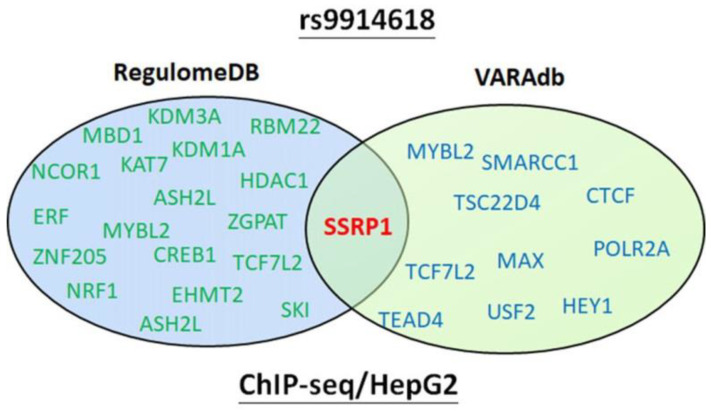
Merging of the rs9914618-associated transcription factor (TF) binding information based on chromatin immunoprecipitation sequencing (ChIP-seq) data obtained from the VARAdb and RegulomeDB databases.

The present study demonstrates that individuals older than 60 years with the mutant base A of rs9914618 had a significantly higher risk of HCC occurrence and LN metastasis under a dominant model (GA + AA). These results were similar to our previous findings, which indicated that the *LINC00673* rs9914618 SNP was linked to the lymphatic spread of oral cancer [27]. Moreover, Zhao et al. also indicated that the *LINC00673* rs9914618 SNP was significantly associated with susceptibility to gastric cancer [16]. SNP variants of an lncRNA were shown to affect its expression or function due to structural changes and to further contribute to cancer progression [28]. Previous reports indicated that rs9914618 is located within the enhancer region containing a CCAAT box [27], the putative binding motif of transcription factors (TFs), including CCAAT/enhancer-binding proteins (C/EBPs) [29], and nuclear transcription factor Y (NF-Y) [30]. NF-Y and C/EBPs were reported to respectively play oncogenic and tumor-suppressive roles in HCC [31,32]. We suggest that rs9914618 polymorphisms may influence interactions with NF-Y and C/EBPs, thereby regulating HCC progression, but this issue should be further investigated in our future work. To further determine the effects of these variations on TF binding, we used variation annotation databases including RegulomeDB and VARAdb [33], and rs9914618-associated TF binding information was based on CHIP-seq data. Both databases showed that rs9914618 affected the binding of the TF termed structure-specific recognition protein 1 (SSRP1), in HCC cells (Figure 3). SSRP1 was reported to promote the proliferation and metastasis of HCC cells, and its upregulation in HCC tissues was correlated with higher T stages and shorter OS times [34], suggesting that rs9914618 variants may impact SSRP1 binding to modulate the progression of HCC. Actually, our present study has indicated that HCC cells carrying rs9914618 AA genotype expressed higher *LINC00673* levels compared to cells carrying the GG genotype, suggesting that the A allele of rs9914618 may produce an increase in *LINC00673* levels in HCC to promote its progression.

Rs11655237 is a common noncoding transcript variant of *LINC00673,* and this genetic variation was investigated in different cancer types, but the results are still controversial. For example, studies showed that the *LINC00673* rs7214041 polymorphism was significantly associated with the development of pancreatic cancer, neuroblastomas, and hepatoblastomas in Chinese populations [18,19,20]. In contrast, this SNP was not correlated with the susceptibility to pediatric gliomas or Wilms tumors in the same ethnic group [35,36]. In addition to Chinese populations, two GWASs demonstrated that rs11655237 SNP could increase the risk of pancreatic cancer in North American, Central European, Australian, and American Jewish populations, but a GWAS of women of European and African ancestry showed that this SNP was not correlated with susceptibility to breast cancer [37,38,39]. These results implied that different clinical impacts of rs11655237 on cancers may be due to different cancer types or ethnicities. In the present study, we observed that rs11655237 SNPs were not correlated with the predisposition to HCC in a Taiwanese population, but further exploration of this genetic factor in relation to HCC will require a larger sample size to verify the current findings.

## 4. Materials and Methods

### 4.1. Study Populations, Ethics, and Consent

HCC patient samples (N = 783) were collected from the National Biobank Consortium of Taiwan (NBCT) and Chung Shan Medical University Hospital (Taichung, Taiwan). In total, 1197 age-, gender-, and ethnicity-matched healthy controls were randomly selected from the Taiwan Biobank Project. All HCC patients had been pathologically confirmed and clinically staged according to the tumor, node, metastasis (TNM) staging system of the American Joint Committee on Cancer (AJCC). Through interviewer-administered questionnaires, we obtained the information about the history of smoking and alcohol consumption from all the recruited subjects. Before collecting venous blood, written informed consent was obtained from each participant, and the investigation protocol was approved by the Institutional Review Board of Chung Shan Medical University Hospital (IRB no. CS2-19133).

### 4.2. Genomic DNA Extraction from Blood

Whole-blood samples from all recruited subjects were collected and placed in ethylenediaminetetraacetic acid (EDTA)-containing tubes. Blood samples were immediately centrifuged to separate genomic DNA from buffy coats, which were isolated by using a QIAamp DNA Blood Mini Kit (Qiagen, Valencia, CA, USA) as previously described [40]. The quality of the final extracted DNA was checked using a Nanodrop-2000 spectrophotometer (Thermo Scientific, Waltham, MA, USA) and preserved at −20 °C [41].

### 4.3. HCC Cell Lines

The human Mahlavu, PLC5, HCC36, SK-HEP-1, HepG2, and Huh7 HCC cell lines were maintained in minimum essential medium (MEM) or Dulbecco’s modified Eagle medium (DMEM) (Gibco, Waltham, MA, USA) with 10% fetal bovine serum (FBS) and a 1% penicillin, streptomycin and glutamine mixture (Thermo Fisher Scientific, Waltham, MA, USA). All HCC cell lines were incubated in a 5% CO_2_-humidified atmosphere at 37 °C.

### 4.4. Selection of LINC00673 SNPs

We selected rs11655237 because this SNP was reported to be correlated with the risks of different cancer types [42]. Moreover, two other SNPs (rs6501551 and rs9914618) located in LINC00673 were selected based on their functional potential with RegulomeDB scores of <3 obtained from the RegulomeDB database.

### 4.5. Genotyping of LINC00673 SNPs

We performed the TaqMan SNP Genotyping Assay with an ABI StepOnePlus™ Real-Time Polymerase Chain Reaction (PCR) System (Applied Biosystems, Foster City, CA, USA) to determine *LINC00673* SNPs including rs11655237 (C/T) (assay ID: C_345893_20), rs6501551 (A/G) (assay ID: C_29084653_10), and rs9914618 (G/A) (assay ID: C_29971800_10). The final results of the *LINC00673* SNPs were analyzed by SDS version 3.0 software (Applied Biosystems) [27,43].

### 4.6. Extraction of RNA and Reverse-Transcriptase Quantitative Polymerase Chain Reaction (RT-qPCR)

Total RNA was isolated from HCC cell lines using TRIzol reagent (Thermo Fisher Scientific) and amplified as described previously [44]. RT-qPCR was carried out using *LINC00673*-specific primers (forward: AATATTAAACGGTCCAGTCCTACAA; reverse: TAGGACTGCCCATTACAGAGGA) and Hot Firepol EvaGreen qPCR Mix Plus (Solis BioDyne, Tartu, Estonia), according to the manufacturer’s instruction. Fluorescence data of detected genes were normalized to the expression of actin using the 2^−^^ΔΔCT^ method.

### 4.7. Bioinformatics Analysis

RNA sequencing analysis and the visualization platform, Gene Expression Profiling Interactive Analysis 2 (GEPIA2), were applied to determine the prognostic effects of *LINC00673/LINC00511*(*ENSG00000227036*) in different cancer types including HCC. Correlations of *LINC00673/LINC00511* with prognoses were calculated using the median cutoff. GEPIA2 performs data mining based on The Cancer Genome Atlas (TCGA) data. The expression level of *LINC00637*, which also refers to *ENSG00000227036*, and related clinical parameters in HCC patients were obtained from TCGA cohort, which was downloaded using UCSC Xena.

### 4.8. Statistical Analysis

Significant differences in categorical variables and demographic characteristic distributions between HCC patients and the healthy controls were determined using the Mann–Whitney U-test. Associations of *LINC00673* genotypes with HCC susceptibility were determined using multiple logistic regression methods and were adjusted for potential confounders such as gender, age, cigarette smoking, and alcohol consumption. Differences in *LINC00673* levels between normal and HCC tissues or in different clinical stages of HCC tissues obtained from TCGA were compared by an independent *t* test. The Statistical Analytical System (SAS Institute, Cary, NC, USA) software (vers. 9.1) was used to analyze all data, and a *p* value of <0.05 was considered significant.

## 5. Conclusions

At present, most HCC patients will eventually develop advanced disease and the treatment outcomes in these patients remain unsatisfactory. However, we still do not have reliable tools to predict who those patients are. Our present study indicated that elevated *LINC00673* expression levels contribute to the development of advanced stages in HCC patients. We found HCC cells carrying rs9914618 AA genotype may cause an increase level of *LINC00673*. We first identified the diverse allelic effects of *LINC00673* SNPs (rs9914618) which contribute to the susceptibility and LN metastasis of HCC in a Taiwanese population. These findings contribute to a better understanding of the risks and early detection of HCC.

## Figures and Tables

**Table 1:** Distributions of demographic characteristics in 1197 controls and 783 patients with hepatocellular carcinoma (HCC).

Variable	Controls (*N* = 1197)	Patients (*N* = 783)	*p* Value
Age (years)	59.41 ± 7.08	62.73 ± 11.70	
<60	479 (40.0%)	298 (38.1%)	*p* = 0.383
≥60	718 (60.0%)	485 (61.9%)	
Gender			
Male	837 (69.9%)	542 (69.2%)	*p* = 0.739
Female	360 (30.1%)	241 (30.8%)	
Cigarette smoking			
No	726 (60.7%)	456 (58.2%)	*p* = 0.284
Yes	471 (39.3%)	327 (41.8%)	
Alcohol consumption			
No	1028 (85.9%)	509 (65.0%)	*p* < 0.001 *
Yes	169 (14.1%)	274 (35.0%)	
HBsAg			
Negative	1051 (87.8%)	517 (66.0%)	*p* < 0.001 *
Positive	146 (12.2%)	266 (34.0%)	
Anti-HCV			
Negative	1144 (95.6%)	515 (65.8%)	*p* < 0.001 *
Positive	53 (4.4%)	268 (34.2%)	
Stage			
I + II		570 (72.8%)	
III + IV		213 (27.2%)	
Tumor T status			
T1 + T2		576 (73.6%)	
T3 + T4		207 (26.4%)	
Lymph node status			
N0		761 (97.2%)	
N1 + N2 + N3		22 (2.8%)	
Metastasis			
M0		753 (96.2%)	
M1		30 (3.8%)	
Vascular invasion			
No		502 (64.1%)	
Yes		281 (35.9%)	
Liver cirrhosis			
Negative		321 (41.0%)	
Positive		462 (59.0%)	

Mann–Whitney U test or Fisher’s exact test was used between healthy controls and patients with HCC. * *p* value of <0.05 was statistically significant. HBsAg, hepatitis B surface antigen; HCV, hepatitis C virus.

**Table 2:** Genotyping and allele frequencies of *LINC00673* single-nucleotide polymorphisms (SNPs) in hepatocellular carcinoma (HCC) patients and normal controls.

Variable	Controls (*N* = 1197) (%)	Patients (*N* = 783) (%)	OR (95% CI)	AOR (95% CI) ^a^
**rs9914618**				
GG	759 (63.4%)	480 (61.3%)	1.000 (reference)	1.000 (reference)
GA	385 (32.2%)	269 (34.4%)	1.105 (0.911–1.340)	1.130 (0.724–1.381)
AA	53 (4.4%)	34 (4.3%)	1.014 (0.650–1.584)	0.936 (0.589–1.488)
GA + AA	438 (36.6%)	303 (38.7%)	1.094 (0.908–1.317)	1.051 (0.954–1.158)
**rs6501551**				
AA	895 (74.8%)	599 (76.5%)	1.000 (reference)	1.000 (reference)
AG	275 (23.0%)	166 (21.2%)	0.902 (0.725–1.122)	0.892 (0.711–1.119)
GG	27 (2.2%)	18 (2.3%)	0.996 (0.544–1.825)	0.822 (0.435–1.551)
AG + GG	302 (25.2%)	184 (23.5%)	0.910 (0.738–1.124)	0.941 (0.843–1.049)
**rs11655237**				
CC	761 (63.6%)	497 (63.5%)	1.000	1.000
CT	388 (32.4%)	260 (33.2%)	1.026 (0.846–1.245)	0.989 (0.809–1.210)
TT	48 (4.0%)	26 (3.3%)	0.829 (0.508–1.354)	0.714 (0.428–1.194)
CT + TT	436 (36.4%)	286 (36.5%)	1.004 (0.833–1.211)	0.979 (0.888–1.079)

OR, odds ratio; AOR, adjusted odds ratio; CI, confidence interval. ^a^ Adjusted for the effects of age, gender, cigarette smoking, and alcohol consumption.

**Table 3:** Genotyping and allele frequencies of *LINC00673* single-nucleotide polymorphisms (SNPs) in hepatocellular carcinoma (HCC) patients and normal controls aged ≥60 years.

Variable	Controls (*N* = 718) (%)	Patients (*N* = 485) (%)	OR (95% CI)	AOR (95% CI) ^a^
**rs9914618**				
GG	476 (66.3%)	293 (60.4%)	1.000 (reference)	1.000 (reference)
GA	211 (29.4%)	174 (35.9%)	**1.340 (1.046–1.717)** ***p* = 0.021**	**1.328 (1.036–1.703)** ***p* = 0.025**
AA	31 (4.3%)	18 (3.7%)	0.943 (0.518–1.717)	0.913 (0.501–1.703)
GA + AA	242 (33.7%)	192 (39.6%)	**1.289 (1.015–1.637)** ***p* = 0.037**	**1.129 (1.002–1.273)** ***p* = 0.047**
**rs6501551**				
AA	535 (74.5%)	374 (77.1%)	1.000 (reference)	1.000 (reference)
AG	167 (23.3%)	103 (21.2%)	0.882 (0.668–1.166)	0.877 (0.663–1.161)
GG	16 (2.2%)	8 (1.6%)	0.715 (0.303–1.688)	0.702 (0.297–1.661)
AG + GG	183 (25.5%)	111 (22.9%)	0.868 (0.662–1.137)	0.928 (0.811–1.063)
**rs11655237**				
CC	446 (62.1%)	310 (63.9%)	1.000	1.000
CT	248 (34.5%)	161 (33.2%)	0.934 (0.731–1.194)	0.917 (0.717–1.174)
TT	24 (3.4%)	14 (2.9%)	0.839 (0.427–1.648)	0.812 (0.412–1.599)
CT + TT	272 (37.9%)	175 (36.1%)	0.926 (0.729–1.175)	0.953 (0.845–1.075)

OR, odds ratio; AOR, adjusted odds ration; CI, confidence interval. ^a^ Adjusted for the effects of gender, cigarette smoking and alcohol drinking.

**Table 4:** Odds ratios (ORs) and 95% confidence intervals (CIs) of the clinical status and *LINC00673* rs9914618 genotypic frequencies in hepatocellular carcinoma (HCC) patients.

Variable	Genotypic Frequencies			
	GG (*N* = 480)	GA + AA (*N* = 303)	OR (95% CI)	*p* Value
Clinical stage				
Stage I/II	350 (72.9%)	220 (72.6%)	1.00	*p* = 0.925
Stage III/IV	130 (27.1%)	83 (27.4%)	1.016 (0.735–1.403)	
Tumor size				
T1 + T2	353 (73.5%)	223 (73.6%)	1.00	*p* = 0.986
T3 + T4	127 (26.5%)	80 (26.4%)	0.997 (0.720–1.382)	
Lymph node metastasis				
No	471 (98.1%)	290 (95.7%)	1.00	*p* = 0.073
Yes	9 (1.9%)	13 (4.3%)	2.346 (0.990–5.557)	
Distant metastasis				
No	464 (96.7%)	289 (95.4%)	1.00	*p* = 0.361
Yes	16 (3.3%)	14 (4.6%)	1.405 (0.676–2.921)	
Vascular invasion				
No	305 (63.5%)	197 (65.0%)	1.00	*p* = 0.675
Yes	175 (36.5%)	106 (35.0%)	0.938 (0.694–1.266)	
HBsAg				
Negative	317 (66.0%)	200 (66.0%)	1.00	*p* = 0.992
Positive	163 (34.0%)	103 (34.0%)	1.002 (0.739–1.357)	
Anti-HCV				
Negative	308 (64.2%)	207 (68.3%)	1.00	*p* = 0.233
Positive	172 (35.8%)	96 (31.7%)	0.830 (0.612–1.127)	
Liver cirrhosis				
Negative	193 (40.2%)	128 (42.2%)	1.00	*p* = 0.573
Positive	287 (59.8%)	175 (57.8%)	0.919 (0.687–1.231)	

The ORs with their 95% CIs were estimated by logistic regression models. HBsAg, hepatitis B surface antigen; HCV, hepatitis C virus.

**Table 5:** Odds ratios (ORs) and 95% confidence intervals (CIs) of the clinical status and *LINC00673* rs9914618 genotypic frequencies in hepatocellular carcinoma (HCC) patients aged ≥60 years.

Variable	Genotypic Frequencies			
	GG (*N* = 293)	GA + AA (*N* = 192)	OR (95% CI)	*p* Value
Clinical stage				
Stage I/II	215 (73.4%)	141 (73.4%)	1.00	*p* = 0.989
Stage III/IV	78 (26.6%)	51 (26.6%)	0.997 (0.660–1.505)	
Tumor size				
T1 + T2	217 (74.1%)	143 (74.5%)	1.00	*p* = 0.918
T3 + T4	76 (25.9%)	49 (25.5%)	0.918 (0.978–1.484)	
Lymph node metastasis				
No	289 (98.6%)	182 (94.8%)	1.00	*p* = 0.013 *
Yes	4 (1.4%)	10 (5.2%)	3.970 (1.227–12.845)	
Distant metastasis				
No	283 (96.6%)	181 (94.3%)	1.00	*p* = 0.220
Yes	10 (3.4%)	11 (5.7%)	1.720 (0.716–4.132)	
Vascular invasion				
No	194 (66.2%)	118 (61.5%)	1.00	*p* = 0.285
Yes	99 (33.8%)	74 (38.5%)	1.229 (0.842–1.794)	
HBsAg				
Negative	225 (76.8%)	144 (75.0%)	1.00	*p* = 0.651
Positive	68 (23.2%)	48 (25.0%)	1.103 (0.721–1.686)	
Anti-HCV				
Negative	169 (57.7%)	118 (61.5%)	1.00	*p* = 0.408
Positive	124 (42.3%)	74 (38.5%)	0.855 (0.589–1.240)	
Liver cirrhosis				
Negative	120 (41.0%)	83 (43.2%)	1.00	*p* = 0.620
Positive	173 (59.0%)	109 (56.8%)	0.911 (0.630–1.317)	

The ORs with their 95% CIs were estimated by logistic regression models. * *p* value < 0.05 was statistically significant. HBsAg, hepatitis B surface antigen; HCV, hepatitis C virus.

## Data Availability

The data presented in this study are available on request from the corresponding author.
